# Abstract of the Proceedings of the Buffalo Medical Association, April 2d, 1872

**Published:** 1872-04

**Authors:** Leon F. Harvey

**Affiliations:** Secretary


ART. III.—*Abstract of the Proceedings of the Buffalo Medical Association. April 2d*, 1872.

Meeting called to order by the President, Dr. Johnson.

Members present: Drs. Miner, Samo, Daggett, Phelps, Strong, Cronyn, Bailey, Sloan, Diehl, Walsh, L. F. Harvey.

This being the Annual meeting the reports of the Secretary and Treasurer were read and accepted. The election of officers for the ensuing year resulted as follows:

*President*—Dr. John Cronyn.

*Vice President*—Dr. John Hauenstein.

*Secretary*—Dr. Leon F. Harvey.

*Treasurer*—Dr. B. H. Daggett.

*Librarian*—Dr. J. B. Samo.

Dr. T. M. Johnson, the President, read the following valedictory address.

Gentlemen.—It is expected of me, as your retiring President, to address you on this occasion in accordance with an established custom of this Association. In fulfilling this obligation it is proper that we first consider the present condition of the Association and its history for the year just past. After which we beg leave to call your attention briefly to some thoughts on the sanitary condition of Buffalo.

The membership of our Association remains about the same as at the commencement of the year; the new members being about equal in number to the loss by death removals from the City and other causes.

There have been twelve regular meetings during the year, and one special meeting. But 'tis matter for regret that the attendance has been much less than it should have been. Much less than the interests of the Association, and the interests of the profession in our midst demands. A painful duty compels me to state that during the year we have lost one of our members by death, viz Dr. B. T. Whitney, who died on the 28th day of Jan. 1872 of Softening of the Brain. Within the year arrangements have been made with the Society of Natural Sciences by which the appearance and comfort of our place of meeting has been much improved and our expenses therefor reduced to about one half their amount heretofore. Our financial condition has been stated to you by our Treasurer. We sincerely hope that in the future the members of the profession in our City will take a more active interest in the Association and by their united efforts make it what it should be.

The subject of the sanitary condition of our City, has of late perhaps become somewhat hackneyed, and some, there undoubtedly are in our midst, who are ready to exclaim ; pray let us hear no more of filth and bad odors.

Still when we remember that during the last two or three years, all our newspapers have been nobly doing their utmost, our Common Council, listening to and discussing all the numerous plans brought to its notice, and the people of all classes clamoring for the abatement of a single nuisance, it is apparent that there is much that might and *ought*, to be said about our general Sanitary condition. The Hamburg canal alluded to above is such a stupendous nuisance as to make it a disgrace to any City in Christendom. There now seems to be a *possibility* that it will be abated within the present year. It is greatly to be desired that it be done at the earliest possible moment. This is desirable because it undoubtedly generates and intensifies disease in its vicinity, is a source of great danger to the general health of the City, and *also*, because it overshadows, and diverts the attention of the public, from other nuisances, other causes of disease and death, which although not so apparant to the general public, are really more deleterious to the health of our City than the Hamburg Canal of which so much has been said. There are within a not very wide extend of territory extending along either side of this same Canal, in a thickly, populated district, scores, yes, hundreds of privies with vaults, many of them at times overflowing and unconnected with any sewer or other adequate means of drainage. Pig-stys, stables, manure heaps, cellars containing water and decaying vegetable mater, cisterns and cess-pools, left from years end to years end, without proper disinfection to contaminate and poison the air, generate disease, and prepare the most inflammable fuel to feed and intensify the first epidemic that makes its appearance among us. This condition of things is not confined to the locality above mentioned, but exists in many other localities, and in some, perhaps to greater extent. This is especially the case in some of the German districts in the easterly portion of the City, where the combined effects of bad drainagel, filth, and a want of knowledge of, and in attention to, Hygienic laws are plainly seen in the prevalence of typhoid disease and the large presentage of child-death that occurs there. The sewerage of large sections of the City is defective and in many instances entirely inadequate. It has many times been proven in other cities, that defective or insufficient drainage is the direct cause of disease and death.

This hygienic law prevails here as elsewhere, and unless the remedy is applied our citizens must suffer the penalty. Another fruitful source of both physical and moral disease, with which the physician comes in daily contact, and of which the general public knows but little, and cannot therefore fully appreciate, is the tenament houses of which this city has its full complement. Unfortunately we have no statistics showing the number and population of these houses. Their number is undoubtedly greater than is generally supposed. In the construction of many of these buildings nearly all the requisites to the health and comfort of the occupants seem to have been dispensed with, and well known hygienic laws disregarded. Their surroundings in some instances are worse if possible than their construction and internal arrangement. Their Areas, Courts and Alleys, with defective drainage are the recepticles of mud, garbage, filth, in short all the abominations that decaying animal and vegetable matter can produce. This material in abundance is left for days and weeks undisturbed by our health authorities to putrify and generate miasma that render them liable at any time to become the centres from which pestilence may extend in all directions.

The State Board of Health of Massachusetts is doing a noble work for the sanitary condition of that State. In the Anual report for 1871 just published, after commenting upon the reports of the causes of typhoid fever, made by the most eminent physicians residing in various cities and villages of that state, the board says. "The conditions and surroundings of typhoid fever in the period of its commencement are now more closely studied than ever before."

"The general result of this study, on the opinions of the Medical World, has been to encourage the belief, that in some way typhoid fever and filth stand in certain relations. There are differences of opinion, but out of this very widely diffused impression, have grown various hypotheses, based upon the propagation of typhoid fever by a poison as definite as that which causes vaccine disease, and all seeking to explain the nature of this poison and the manner of its introduction into the healthy human body. They may be thus divided."

First—Propagation by drinking water made foul by the decomposition of organic matter, whether animal or vegetable, and especially by the presence in such water of excrementitious matter discharged from the bodies of those suffering from typhoid fever.

Second—Propagation by air contaminated by any form of filth, and especially by privies, cesspools, pig-stys, manure heaps, rotten vegetables in cellars, leaking or obstructed drains.

Third—Emanations from the earth, occurring especially in the autumnal months and seasons of drouth.

How exactly these conditions correspond with the conditions in and around the tenament houses of which we have just spoken. The moral condition of many of the truly unfortunate occupants of these houses, is as bad as their physical condition. It is entirely unnecessary for the curious, the philanthropist or the good samaritan to go in imagination with Dickens through the alleys and byways of London to White Friars and Tom All-alones, to see abject poverty, misery, and vice, with all its loathsome surroundings. All these evils exist to a fearful extent in our midst, and within a few minutes walk of pallatial residences. The occupants of these modern palaces, and all of the better portion of our citizens owe it to their own safety, and the safety of those dear to them and depending upon them, to see to it, that in some way the condition of the unfortunate class heretofore alluded to be greatly ameliorated and improved. They ought to, and most of them do know the elevating, christianizing influence of the observance of hygienic laws, of healthful, and beautiful surroundings. They can and it is for their own interests to see to it, that proper health laws are made, and that the proper officers to execute these laws do their whole duty. The people and *ourselves*, have not done our whole duty in this matter.

Milk—Time will not permit us on this occasion to much more than allude to this subject of great sanitary importance. Within the last week with a member of this association who is now investigating this subject, we visited several of the stables in which the cows are kept that supply a part of the milk used in the city. We were much surprised on learning the number of stables and cows kept within the city limits, and much more surprised at the *manner* in which they are kept. Their natural and healthy condition, appears for the most part, to be entirely disregarded. They are crowded together in numbers varying from two to thirty, in dark, damp, and filthy stables, and deprived of adequate exercise, pure air, and pure water, for months, in some instances we are informed for years. Many of them are fed almost entirely upon still slop and when sufficiently supplied with this kind of food may yield a plentifully supply of milk. But it is well known to the initiated that it is so deficient in the proper essentials to good milk that it scarcely deserves the name of milk at all. Swill milk is not an inappropriate name for it. The gentlemen referred to in this connection informs us that the milk retailed to consumers is often adulterated with from twenty to twenty-five per cent. of water and contains only from three to six per cent. cream. Good country milk contains from fifteen to eighteen per cent of cream. Is it strange, that so many infants and children die of cholera infantum and kindred diseases, when dependent upon this food for sustenance. The venders of this fluid pursue the even tenor of their way with not the slightest interference by our health authorities We hope that the gentleman investigating this subject will in some manner publish his report for it is a subject of great interest.

Small Pox—It seems entirely unnecessary to say anything of the desirability of protecting every person from this loathsome disease. It is the duty of every person who does not know that himself and all in whom he is interested are protected to resort at once to vaccination, Yet we believe there are many amongst us that are not thus protected. It is to be feared that the mode of vaccination in some instances resorted to by our health authorities does not secure protection.

We were told a few days ago, by a teacher, a gentleman long connected with our public schools, that in one department of one of the schools, one hundred, being all the scholars in the department, were vaccinated and not one of them took the vaccine disease. From our own observation, and from what we learn from physicians and teachers, we are compelled to believe that vaccination as done in our public schools, is a failure, and a waste of the peoples money. We find no fault with the gentlemen who do these services. We believe them capable and faithful. The fault lies in the system adopted by our health authorities. There is need of stringent laws regulating this matter of vaccination.

It was our intention at the outset to ascertain and place before you if possible, the effects of the Hamburg Canal upon the health of people living in its vicinity. But on going to the health office for information on this subject, we soon found ourselves in the pursuit of knowledge under difficulties. The only records there that would at all aid in the investigation consist in copies of the certificates of cause of death given by physicians, undertakers, and coroners. The information to be thus gained was considered so unreliable, and attended with so much labor, that the project was abandoned. We were surprised and chagrined to find in the course of these investigations that the Board of Health had not made a printed annual report for the last ten years. Such a report, if properly made, would be valuable both to the profession and the public. Hoping that it may be of interest to members of the profession, the following condensed mortuary report for the year 1871 is placed before you.

The whole number of deaths reported to have occurred in the city during the year is 1635. In addition to these, 68 still born are reported. Of the whole number of deaths 892 were males and 734 were females. 1061 were reported as native born 548 foreign born and 26 unknown. 289 were of American parentage 1161 of foreign parentage and 185 parentage unknown. 428 are reported married 1120 single 87 unknown. 1544 died in the city at large and 91 in hospitals and other public institutions. From one hospital containing a daily average of about one hundred patients, no deaths are reported as occurring. The attending physician has reported two deaths during the year. Several other deaths have occurred there and have probably appeared in the reports, as in the city at large and cause unknown. This state of things is not the fault of the physician in charge. 86 died before attaining the age of thirty days. 155 died at the age of from one month to six months, 111 from 6 to 12 months, 224 from one year to three years. 81 from 3 to 5, and 39 from 5 to 10 years. The number of certificates of death given by physicians is 1118. By undertakers 366, by coroners 151. Consumption claims the largest number of decedents 205. Deaths from some of the other causes occurred as follows: Accidents 70, disease of the heart 58, cholera infantum 54, croup 84, inflammation of the brain 29, brain and meningies 30, inflammation of the lungs 84, typhoid fever 102, scarlet fever 34, puerperal fever 14, inflammation of the bowels 29, marasmus 20, diptheria 38, General dropsy 21, dropsy of the brain 15, of old age 42, from causes reported as unknown 317. Estimating the population at 125,000 which is nearly correct, the death rate for the year was one in 76.4 of the population or 13 in a thousand. Of the decedents, about 1 in 5 died before reaching the age of one year and 1 in 4 before the age of 5 years, 1 in 23 are reported as stillborn, 1 in 8 died of consumption, and 1 in 16 of typhoid fever. We could go much further into these particulars, but believe the reports too unreliable, to be of much if any value. Their unreliability is in some instances very apparant. We know at a glance that the number 68 does not probably represent more than one third the number of still-born. The number reported as dying from causes unknown is large, and as statistics, are of no value to us. This is to some extent the fault of physicians who do not always write certificates of cause of death. At no time during a residence of ten years have we seen the sanitary condition of this city, so bad as it is now, and at no period during that time, have we been more likely to suffer from epidemic diseases, than during the coming summer. There is great need of reform in the health department of our city government. This fact must be evident to any observing person who walks our filthy streets. It is the duty of every practitioner of medicine, among us to use his influence for reform in this matter.

In conclusion gentlemen. As physicians, we shall not fulfill our whole mission unless we use our best endeavors to *prevent disease* as well as to paliate and cure the sick.

Dr. Miner—The paper just read is an important one, and we have all been very much interested in it. The Doctor says the city is in a worse condition than ever before, but has there been time to get the streets cleaned up earlier? The out houses are no worse than ever, always bad enough. He is justified in saying some hard thing about the Hamburg canal, but I think the Doctor cannot show that it has been the direct cause of disease, there has been no case in its vicinity that has not occurred in other parts of the city. I think I am safe in saying this. The canal has not been spoken of too highly as a nuisance or a disgrace. The paper will and should attract much attention. Our sanitary condition should be understood, if not abated. It is astonishing, how impossible it is to get proper drainage in our best streets. The houses may look well on the outside but there is not, and cannot be health inside with improper drainage. In the warm season in Buffalo, there is hardly a neighbourhood where on the house tops, effluvia from the out houses may not be observed.

Dr. Strong—There is a wide field for observation in the paper just read. We should as a society look to the sanitary condition of the city, there should be an annual report made by the Health Physician treating on this subject. Every medical man feels the need of it, and I would therefore move that a committee be appointed to confer with the proper authorities on the importance of publishing such a report.

Motion carried.

The President appointed as such committe Drs. Strong, Rochester and Miner.

Dr. Johnson—There has been no annual report of the Health Physician for eight or ten years. Smaller cities than ours have good reports presented every year.

Dr. Daggett—I was ill at the time of my appointment to the office of Health Physician, and was some time before I could get fairly at work. Found the mortuary law of the city defective. Visited the different cemeteries in our city, and after thorough study of the subject in all its bearings, succeeded with great difficulty in getting a revision of the City Ordinances. Received no encouragement to do anything. Physicians are very careless in giving death certificates. Undertakers said frequently they could not get certificates. Physicians are liable to a fine if called upon for one and refuse to give it.

The appointment of Health Physician being every year, it is difficult for one holding the office for so short a time, to do justice to it, for just as he becomes conversant with its duties, he steps out to let another learn them. While in office found it very difficult to enforce the law.

Dr. Johnson—If the Hamburg canal is not now detrimental to health, it will be in the summer or in case of an epidemic.

The nuisances in other parts of the city are worse than ever. In the vicinity of William street the city has built up very rapidly and there is no drainage there. It will be hard to resist an epidemic there. Do not think that people think enough of drainage.

Dr. Cronyn said that the subject of cerebro-spinal meningitis had been mentioned and deferred, Dr. Miner had come in and would have something to say upon the subject.

Dr. Miner said he had little to add to what he had attempted to say in the last Journal concerning this malady. Every day he had seen new cases and believed the disease on the increase. It commenced with children, but adults are now frequent victims, though it is still mostly confined to children and young people. Its appearance in Buffalo opens up a wide field for professional labor and also professional research and study. At present we cannot be supposed to possess a great amount of independent knowledge, but must trust to the observations of others for our views of the nature of the disease. It would seem that we are justified in saying, that it is epidemic, *not contagious.* This question is asked constantly and is to be answered positively. Its causes we do not understand. They are supposed to be atmospheric and wide spread, but of this, and the nature of this influence we know nothing. The symptoms and appearences are now sufficiently familiar to all physicians. The proportion of deaths to cases could not yet be determined. but it seems probable that a large majority recover. *Treatment* was to us the most important subject. Opium and Quinine he thought proper, the opium in many cases indispensable. He had used chloral to obtain sleep, and liked its effects, cases recovered under all plans of medication, suggesting to the observing practitioner that like many other diseases it might have self limited nature, and that the final result might not be greatly influenced by drugs; that they are useful mainly in the *care* of patients. Many remedies had been, and would continue to be tried; care is required to determin their real value.

Dr. Strong—Have seen very little of this affection since my return to the city, only three or four cases, two children, in the same family, one died, in the fatal case there was not marked delerium, much pain, rigidity of the muscles of the neck, no coma, last five or six hours might have been comatose. Am now treating a lady æt 35 taken Saturday with chill, prostration to fainting, pain in head and back for thirty six hours, after that the chief pain seemed to be in the lower extremeties, rigidity of the muscles of the neck no coma, no delerium, the pain was very severe, the patient said that it seemed as if her legs would burst, the friends said that on the first day there were spots on the body. Gave opium very freely the first forty eight hours $\frac{3}{4}$ gr. to 1 gr. also used the bromide of potassium, mustard on the neck, gave stimulants. The patient is now apparently out of danger. Have the impression that coma and delerium is not common, would ask the views of the gentlemen present. Do not think that spots are characteristic.

Dr. Miner—Delerium must be a common symptom of the disease, patients are frequently insensible to every thing.

Dr. Cronyn moved that this disease be made the subject for discussion at the next meeting. Carried.

Dr. Cronyn—I think that the delerium has arisen from the pain in most of the cases I have seen, there is a tetanic condition of the muscles, opisthotonos. Adjourned.

Leon F. Harvey, Secretary.

